# A new monoclonal antibody (CAL2) detects CALRETICULIN mutations in formalin-fixed and paraffin-embedded bone marrow biopsies

**DOI:** 10.1038/leu.2015.192

**Published:** 2015-08-14

**Authors:** H Stein, R Bob, H Dürkop, C Erck, D Kämpfe, H-M Kvasnicka, H Martens, A Roth, A Streubel

**Affiliations:** 1 Reference and Consultation Center for Lymphoma and Haematopathology, Pathodiagnostik Berlin, Berlin, Germany; 2 Synaptic Systems GmbH, Göttingen, Germany; 3 Praxis für Onkologie, Lüdenscheid, Germany; 4 Senckenbergisches Institut für Pathologie, Johann Wolfgang Goethe-Universität, Frankfurt am Main, Germany; 5 Medizinisches Versorgungszentrum am Helios Klinikum Emil von Behring, Labor für molekulare Diagnostik und Mikrobiologie, Berlin, Germany

## Abstract

Recent advances in the diagnostic of myeloproliferative neoplasms (MPNs) discovered *CALRETICULIN (CALR)* mutations as a major driver in these disorders. In contrast to *JAK2* mutations being mainly associated with polycythaemia vera, *CALR* mutations are only associated with primary myelofibrosis (PMF) and essential thrombocythaemia (ET). *CALR* mutations are present in the majority of PMF and ET patients lacking *JAK2* and *MPL* mutations. As these *CALR* mutations are absent from reactive bone marrow (BM) lesions their presence indicates ET or PMF. So far these mutations are detectable only by molecular assays. Their molecular detection is cumbersome because of the great *CALR* mutation heterogeneity. Therefore, the availability of a simple assay would be of great help. All *CALR* mutations reported lead to a frameshift generating a new 36 amino-acid C-terminus. We generated a monoclonal antibody (CAL2) to this C-neoterminus by immunizing mice with a representative peptide and compared its performance with Sanger sequencing data in 173 MPNs and other BM diseases. There was a 100% correlation between the molecular and the CAL2 immunohistochemical (IHC) assays. Thus, the detection of *CALR* mutations by the CAL2 IHC is a specific, sensitive, rapid, simple and low-cost method.

## Introduction

Bone marrow (BM) biopsy histology is mandatory for discriminating the different chronic Philadelphia chromosome-negative myeloproliferative neoplasms (MPNs) from reactive BM lesions and from each other. This discrimination is in a proportion of cases not possible on purely histological grounds. The discovery of mutations in *JAK2*, *CALRETICULIN (CALR)* and *MPL* genes has greatly facilitated this differential diagnosis. Polycythaemia vera is associated with *JAK2* mutations *(JAK2 V617F* and *JAK2* exon 12 mutations) in virtually all cases. In contrast, *JAK2* mutations are present in essential thrombocythaemia (ET) and primary myelofibrosis (PMF) in only 50–60%. Mutations of the *thrombopoetin receptor (MPL)* gene are detectable in 3–5% of ET and 5–8% of PMF patients.^1–3^ *JAK2* and *MPL* mutations were selected as the major diagnostic criteria for MPNs in the 2008 World Health Organization (WHO) classification.^4^ Recently, mutations of the *CALR* gene were found in 50–80% of *JAK2* and *MPL* mutation-negative ET and PMF patients.^5, 6^ Because of this high mutation frequency, detection of *CALR* mutations is already widely included in the diagnostic programme for MPN.

So far *CALR* mutations are only detectable by molecular assays. These assays are complicated because of the high heterogeneity of *CALR* mutations with at least 40 different types. These mutations are represented by insertions or deletions, all located in exon 9.^7^ All mutations cause a frameshift, which lead to a unique alternative reading frame coding a novel protein C-terminus consisting of approximately 36 amino acids.^5, 6, 8^ Vannucchi *et al.*^8^ have successfully raised in rabbits a polyclonal antiserum against a peptide containing significant parts of the novel C-terminus of mutated *CALR*. With this antiserum CALR-mutated cells could be detected in formalin-fixed routinely processed BM sections of patients with ET and PMF carrying *CALR* mutations. However, the polyclonal antibody approach provides only a limited amount of antiserum and usually requires affinity purification of the obtained antiserum by the immobilized immunogene. These limitations can be overcome by the monoclonal antibody (mAb) technology.

Here, we report about the generation of a mouse hybridoma designated as CAL2, which secrets antibodies that selectively stain cells carrying mutated *CALR* proteins in routinely processed BM paraffin sections.

## Materials and methods

### Antigen peptide, immunisation and hybridisation

The hybridomas were generated by a standard protocol of Synaptic Systems (Göttingen; see also http://www.sysy.com/mabservice.html) as followed. Briefly, we expressed the novel C-terminus peptide (-KM SPARPRTSCR EACLQGWTEA) of mutated *CALR* in *Escherichia coli* (BL21 D3) as immunogene. Three 8- to 10-week-old BALB/c female mice were subcutaneously immunized over a period of 75 days. Cells from the knee lymph nodes were fused with the mouse myeloma cell line P3X63Ag8.653 (ATCC CRL-1580). The clones used in this study were re-cloned two times by limiting dilution and the immunoglobulin subclass was determined.

### Hybridoma screening

The antibodies secreted by the hybridomas were screened for their reactivity against the immunogene by ELISA. The positive mAbs were retested by immunofluorescence on HEK 293 cells transiently transfected with a pEGFPC2-*CALR*-mutation plasmid, overexpressing the mutated C-terminus of *CALR* (KMSPARPRTSCREACLQGWTEA) fused to the C-terminus of enhanced green fluorescent protein (EGFP), using the Mirus TransIT kit (Madison, WI, USA) according to the manufacturer's instructions. To test the performance of the selected mAbs on paraffin sections of formalin-fixed HEK 293 cells transiently transfected with pEGFPC2-mutated *CALR* and wt HEK 293 cells were stained with the supernatants of the obtained clones using the immunodetection method described below. The clones with the best performance were selected and designated as CAL1, CAL2 and CAL3.

### Human tissue specimen

One hundred and seventy-three specimens including BM samples consisting of myeloid and non-myeloid neoplasms as well as non-neoplastic samples (details in Table 1) were obtained from the archive of the Pathodiagnostik Berlin (Germany), Institute of Pathology of the University Frankfurt (Germany) and from Dr Kämpfe (Lüdenscheid, Germany).

The study was approved by the ethics committee of the University of Frankfurt. All sample evaluations were performed without any knowledge of individual patient characteristics and all samples were strictly anonymized and renumbered. The samples were reviewed by RB, HD, HS and partially by H-MK using the criteria of the 2008 WHO-classification.^4^

### Immunostaining and molecular assay

Immunohistochemical (IHC) staining was performed as recently described by Bob *et al.*^9^ The IHC with the mAbs CAL1, CAL2 and CAL3 was first performed in 20 MPNs molecularly tested for *CALR* mutations, 10 with and 10 without mutation. The mAb with the strongest specific reaction (CAL2, available in Europe at Dianova, Germany and in USA at HistoBioTec, USA) was selected for the investigations of human tonsils and 152 more BM samples (details in Table 1). These stainings were blindly evaluated by HS, RB and HD. We tested the reproducibility of the CAL2 IHC by repeating the CAL2 staining four times on sections of 10 cases with a *CALR* mutation and of 10 cases without a *CALR* mutation. All 173 cases mentioned above were analysed for the presence of mutated *CALR* by Sanger sequencing using nucleic acids extracted from the BM specimens. The found mutations were designated according to the recommendation by the Human Genome Variation Society.

### Statistical analysis

Statistical analysis was performed with the *χ*^2^ test.

## Results

### Selection of the CALR mutation-specific monoclonal clones CAL1, CAL2 and CAL3

**Figure 1:**
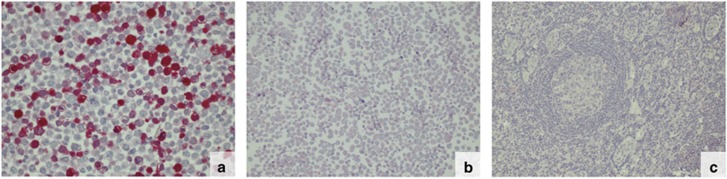
(**a**–**c**) Immunostaining of formalin-fixed, paraffin-embedded HEK 293 cells transfected with the novel C-terminus of mutated *CALR* (**a**) and non-transfected HEK 293 cells (**b**) and a tonsil (**c**) with the antibody CAL2. The antibodies CAL1 and CAL3 produced an identical staining result (magnification: x80).

In total 2300 hybridomas were obtained. By screening the secreted antibodies, three mAbs (CAL1, CAL2 and CAL3) were identified, which demonstrated a specific and selective reaction with the immunogene expressed in transiently transfected HEK 293 cells before and after formalin fixation and paraffin embedment. These three mAbs did neither react with the non-transfected (wild-type) HEK 293 cell lines nor with a human tonsil (Figures 1a–c).

### Comparison of the results obtained by Sanger sequencing and IHC with the antibodies CAL1, CAL2 and CAL3

All three selected mAbs stained specifically the 10 mutated cases but not the non-mutated BM samples. The mAb CAL2 produced the strongest specific reaction and was applied for the staining of the additional 153 cases (in total 173) of MPNs, other BM diseases and normal BMs (Table 1). All 20 repeated CAL2 stainings produced identical results, being positive in the 10 cases carrying *CALR* mutations and being negative in the 10 cases without *CALR* mutations. The comparison of the results of the Sanger sequencing in these 173 cases and IHC with CAL2 showed a 100% correlation (*P*<0.005).

Table 2 provides an overview of the *CALR* mutation genotypes observed in the BM samples of 52 MPN patients with *CALR* mutations detected by Sanger sequencing and CAL2 IHC. These results showed that the CAL2 antibody recognised eight different genotypes, whereby three members of type 1 and 2 mutations were the most frequent ones, accounting for ca 85% of all genotypes. Two of the eight genotypes belong to the rare category. Three of the detected genotypes were not listed in the Human Genome Variation Society. The cases with mutated *CALR* were restricted to PMF, ET and cases in where the discrimination between ET and prefibrotic PMF was not possible.

### Predominant expression of mutated CALR in megakaryocytes

**Figure 2:**
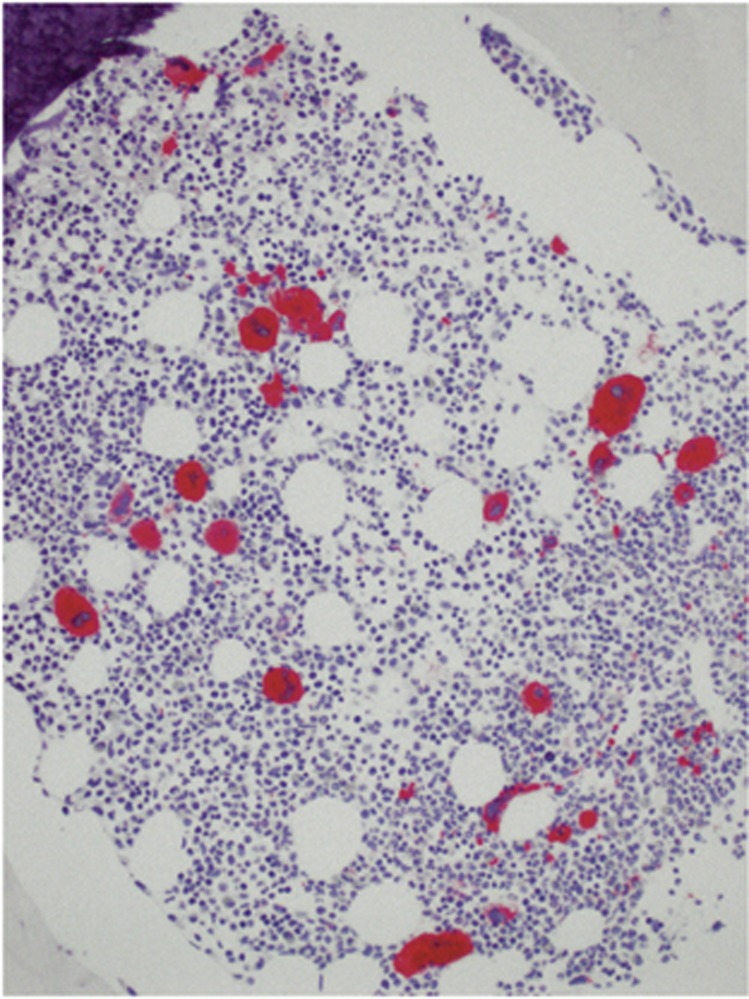
Immunostaining of a formalin-fixed, paraffin-embedded BM biopsy from an ET patient with the mouse monoclonal antibody CAL2 (magnification: x50). The megakaryocytes are strongly labelled. *CALR* mutation was confirmed by Sanger sequencing.

**Figure 3:**
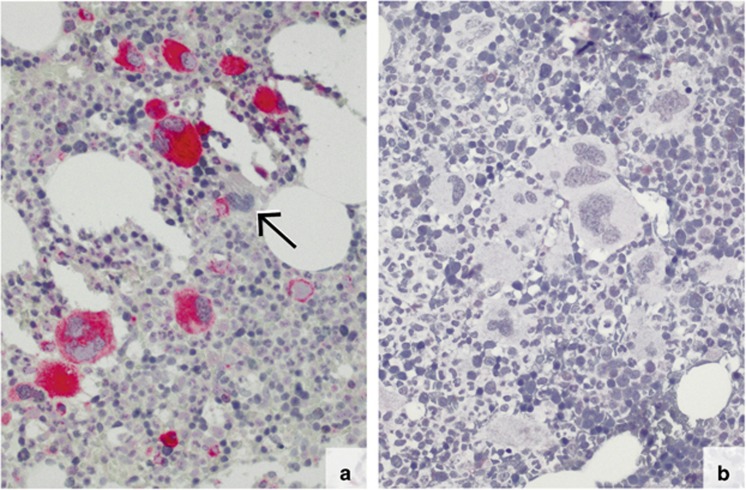
Immunostaining of formalin-fixed, paraffin-embedded BM biopsies from prefibrotic PMF patients with the mouse monoclonal antibody CAL2. Nearly, all megakaryocytes in the case with genotypically demonstrated *CALR* mutation are strongly labelled (**a**; magnification: x200), whereas the megakaryocytes of the case without a *CALR* mutation remained unlabelled (**b**; magnification: x250). The *CALR* mutation status was confirmed by Sanger sequencing. In **a**, an unstained megakaryocyte is marked by an arrow.

**Figure 4:**
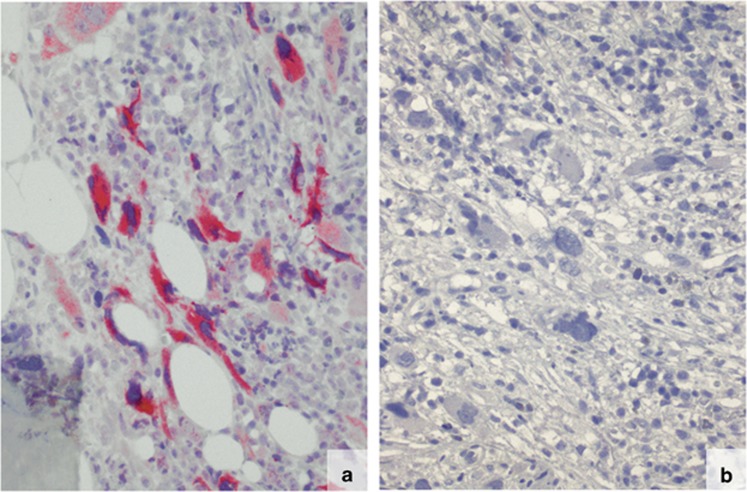
Immunostaining of formalin-fixed, paraffin-embedded BM biopsies from fibrotic PMF patients with the mouse monoclonal antibody CAL2. The megakaryocytes in the case with genotypically demonstrated *CALR* mutation are strongly labelled. The fibres are not labelled (**a**; magnification: x250). The megakaryocytes of cases without a *CALR* mutation remained unlabelled (**b**; magnification: x250). The *CALR* mutation status was confirmed by Sanger sequencing.

**Figure 5:**
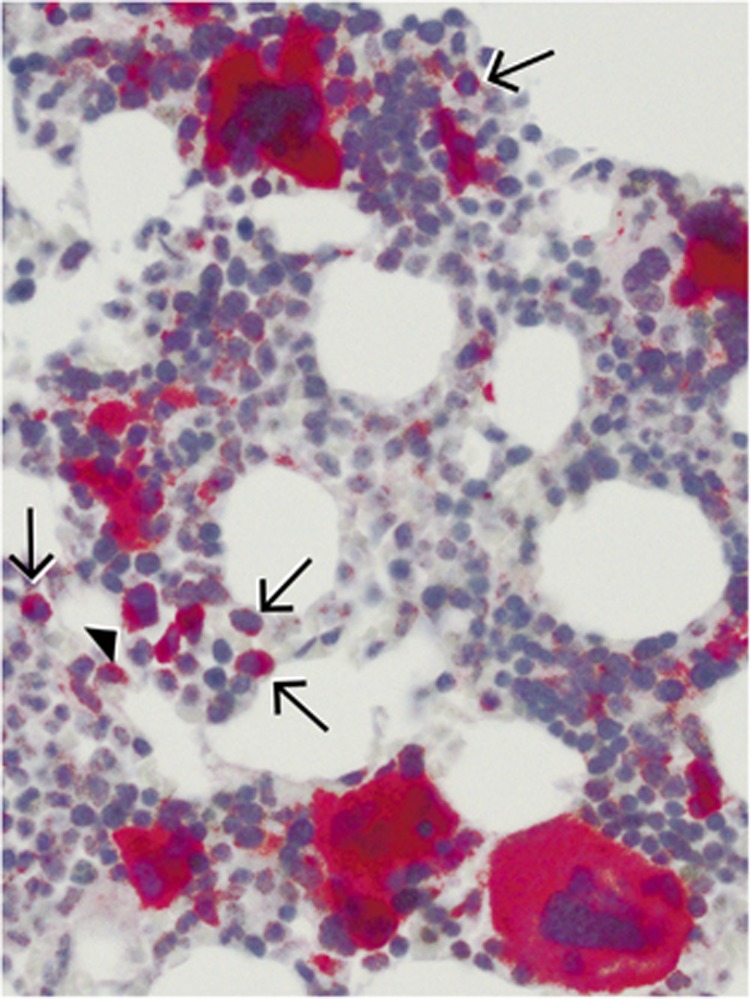
Immunostaining of a formalin-fixed, paraffin-embedded BM biopsy from a PMF patient with the mouse monoclonal antibody CAL2. In addition to the strongly stained megakaryocytes, some smaller cells are labelled (arrowed; magnification: x200).

The CAL2 antibody showed strong immunostaining of more than 90 to 97% of the megakaryocytes in all cases in which Sanger sequencing demonstrated a *CALR* mutation (Figures 2, 3a, and 4a; *P*<0.005). A single unstained megakaryocyte (arrowed) is shown in Figure 3a. The cases without a genotypically detected *CALR* mutation remained totally unlabelled with the CAL2 antibody (Figures 3b and 4b). In samples with fibrosis, the spindle shaped and morphologically deformed megakaryocytes were positive and clearly recognisable (Figure 4a). The fibrotic material remained unstained. In a proportion of samples, few smaller cells were stained by the CAL2 antibody (arrowed in Figure 5). The lineage of these smaller cells could not be clarified in the present study.

## Discussion

Mutations in the *CALR* gene have been discovered in 50–80% of ET or PMF patients without mutations of the JAK2 or MPL genes,^5, 6^ indicating that this discovery is a further important step in the improvement of the diagnostic and characterisation of MPNs and for the application of kinase inhibitor therapy.

The *CALR* gene is located at the short arm of chromosome 19. All known *CALR* mutations are located in exon 9 and represent either somatic deletions or insertions.^7^ The 52-bp deletion (p. L367fs*46) and 5-bp insertion (p.K385fs+47) are the most frequent mutations. The remaining mutations are very heterogeneous. For the detection of all *CALR* mutations in exon 9, molecular genetic assays are required. Their performance time consuming, and technically as well as financially not possible in many medical units for routine diagnostics. Therefore, a simpler, more rapid and more cost-effective method is needed. The development of such a method is possible because of the fact that all exon 9 mutations of the *CALR* gene cause a C-neoterminus of the CALR protein with a minimum of 36 amino acids replacing the normal 27-amino-acid sequence.^5, 6, 8^ To take advantage of the abnormal C-neoterminus peptide embracing all *CALR* mutations, we generated the mAbs CAL1, CAL2 and CAL3 against a peptide representative for the abnormal novel C-terminus. All three antibodies labelled selectively the megakaryocytes in the BM sections from CALR-mutated patients, enabling the differentiation between CALR-mutated and *CALR*-non-mutated patient samples. As the antibodies secreted by the hybridoma CAL2 produced the strongest and cleanest staining, we selected this clone for the investigation of larger number of BM diseases. The data of this investigation demonstrated an absolute correlation between the detection of *CALR* mutations by Sanger sequencing and by CAL2 IHC.

The CAL2 immunostaining pattern obtained in mutated BM sections showed that mutated CALR is strongly expressed in more than 90 to 97% of megakaryocytes. The very few megakaryocytes remaining unstained probably represent residual non-neoplastic ones. The CAL2 antibody staining was negative on the vast majority of smaller cells, that is, erythropoietic or granulopoietic cells. This finding is in keeping with the results of comparative gene expression profiling data showing that the level of the wt *CALR* mRNA in non-mutated samples is approximately five or six times lower in granulopoietic and erythropoietic cells than in megakaryocytes.^8^ These data can explain why the expression level of mutated *CALR* in BM cells other than megakaryocytes is low and appears to be below the threshold of CAL2 IHC.

Vannucchi *et al.*^8^ showed that also wt *CALR* is highly expressed in megakaryocytes indicating that expression pattern of mutated *CALR* resembles the expression profile of the wt *CALR*. In a proportion of cases, the mAb CAL2 moderately labelled some of the smaller cells. Although it is beyond the scope of this study, it is tempting to speculate whether these smaller cells represent small megakaryocytes or neoplastic immature granulopoietic or erythropoietic cells. Cabagnols *et al.*^10^ showed that the CALR allelic burden of neutrophils of peripheral blood was in ET samples partially less than 25% and in many PMF samples less than 60%, which suggests that many granulocytes in ET and PMF samples with *CALR* mutation do not harbour *CALR* mutations and thus appear to be non-neoplastic. This might also explain why only a small number of non-megakaryocytic cells are stained with the CAL2 mAb. Our data demonstrated that megakaryocytes are the group of cells, which express mutated *CALR* in 97% at a high level, that is, about 97% of the megakaryocytes are neoplastic. Owing to multiple internal mitosis, megakaryocytes usually contain more than two chromosomes 19 and harbour a high allele number of CALR. In peripheral blood, it is shown that Sanger sequencing has a relatively low sensitivity for the detection of *CALR* mutation.^11^ The high allele number and the high frequency of neoplastic megakaryocytes compensate this limitation in BM specimens.

The eight genotypes observed in our study are listed in Table 2. They include one sequence of known type 1 mutations and two sequences of the known type 2 mutations detected in 85% of our samples. Two sequences belong to the known rare mutation group and three sequences are not yet listed in the Human Genome Variation Society. These eight sequences include 88% of mutations detected by Kampfl *et al.*^6^ and 87% of mutations described by Nangalia *et al.*^5^ However, it is evident that all different types of *CALR* mutation results in an identical novel C-terminal peptide, specifically recognised by the new CAL2 antibody. Therefore, it can be concluded that the IHC staining with mAb CAL2 is able to detect all known *CALR* mutations, although not all known *CALR* mutations were present in the samples of our study.

Taken together, CAL2 IHC is a more convenient, more rapid and cost-effective method than molecular assays for specific identification of *CALR* mutations in BM specimens. Furthermore, it provides a rapid indication for the application of molecular assays for *JAK2* mutation and in case of need for the clarification whether the *CALR* mutation is of type 1 or type 2 or another variant.^10^

## Figures and Tables

**Table 1:** Correlation between CALR mutations detected by Sanger Sequencing and CAL2-immunohistochemistry in samples obtained from bone marrow of patients with myeloproliferative neoplasms or other disorders and from control tissues

*Disease type*	*No. of samples*	*No. of cases with detected mutations*	
		*Sanger sequencing*	*CAL2 IHC*
MPN NOS	17	12	12
PMF	52	20	20
ET	59	20	20
PV	19	0	0
Myeloid neoplasms other than PV, ET and PMF	8		
RARS-T	1	0	0
MDS with fibrosis	1	0	0
RAEB-1	1	0	0
CNL	1	0	0
CML	1	0	0
aCML	1	0	0
Mastocytosis	2	0	0
BM with non-myeloid neoplasm	8		
CLL	3	0	0
MCL	1	0	0
HCL	1	0	0
PTCL	1	0	0
cHL	1	0	0
MGUS	1	0	0
Non-neoplastic tissue	10		
BM in Iron deficiency	1	0	0
BM in idiopathic thrombocyopenia	1	0	0
Normal BM	4	0	0
Tonsils	4	0	0
Total No	173	52	52

Abbreviations: aCML, atypical chronic myeloid leukaemia, BCR-ABL1 negative; BM, bone marrow; CALR, CALRETICULIN; cHL, classical Hodgkin lymphoma; CLL, chronic lymphocytic leukaemia; CML, chronic myelogenous leukaemia, CNL, chronic neutrophilic leukaemia; ET, essential thrombocythaemia; HCL, hairy cell leukaemia; IHC, immunohistochemistry; MCL, mantle cell lymphoma; MDS, myelodysplastic syndrom; MGUS, monoclonal gammopathy of undetermined significance; MPN NOS, myeloproliferative neoplasm not otherwise specified, that is, MPN cases where the differential diagnosis between prefibrotic PMF and ET was not possible; PMF, primary myelofibrosis; PTCL, peripheral T-cell lymphoma; PV, polycythaemia vera; RAEB-1, refractory anaemia with excess blasts-1, BCR-ABL1 positive; RARS-T, refractory anaemia with ring sideroblasts in transformation.

**Table 2:** Relative frequency of *CALR* mutation types observed in samples from 52 patients with myeloproliferative neoplasms harbouring *CALR* mutations detected by Sanger sequencing and CAL2 immunohistochemistry

*Genotypes*	*Frequency of the genotypes of the studied samples*	*Frequency of the genotypes according to Klampfl et al.*	*Frequency of the genotypes according to Nangalia et al.*
	*(%)*	*(%)*	*(%)*
Total	52	150	147
			
*Type 1*			
L367fs*46	27 (52)	67 (44.7)	67 (45.5)
			
*Type 2*			
K385fs*47	15 (28.8)	65 (43)	61 (41.5)
D373fs*51^&^	2 (3.8)	0	0
			
*Rare genotypes*			
L367fs*52	3 (5.8)	1 (0.7)	1 (0.7)
L367fs*48	1 (1.9)	2 (1.4)	2 (1.4)
E406del^a^	2 (3.8)	0	0
K375fs*49^a^	1 (1.9)	0	0
E370fs*38^a^	1 (1.9)	0	0

a Genotypes so far not listed in Human Genome Variation Society, the genotype marked with & is described in Cabagnols *et al.*^10^^&^Marker.
